# Does arts and cultural engagement vary geographically? Evidence from the UK household longitudinal study

**DOI:** 10.1016/j.puhe.2020.04.029

**Published:** 2020-08

**Authors:** H.W. Mak, R. Coulter, D. Fancourt

**Affiliations:** a Department of Behavioural Science and Health, University College London, 1-19 Torrington Place, London, WC1E 7HB, UK; b Department of Geography, University College London, Pearson Building, Gower Street, London, WC1E 6 B, UK

**Keywords:** Arts and cultural participation, Geographical factors, Geographical and health inequalities, Spatial setting, Neighbourhood characteristics

## Abstract

**Objectives:**

Previous studies have shown the beneficial impacts of arts participation and cultural engagement on health outcomes. However, this engagement is socially patterned and is also possibly influenced by geographical factors.

**Study design:**

The aim of this study was to examine the association between geographical factors (spatial setting and neighbourhood characteristics) and arts and cultural engagement amongst adults in the UK.

**Methods:**

Data analysed were from Understanding Society Wave 2 (2010/12) with a total sample size of 26,215. Logistic and ordinal regression was used to identify geographical predictors for the patterns of the engagement.

**Results:**

Our results show that there are geographical differences in participation independent of individual demographic and socio-economic backgrounds. In particular, there was more evidence for differences in the participation based on neighbourhood characteristics (e.g. level of area deprivation). We also found some interactions between individual and geographical factors for cultural engagement but not for arts participation.

**Conclusions:**

This study reveals a geographical and individual socio-economic gradient in arts and cultural engagement. Given the health benefits of arts engagement, improving access to arts and cultural programmes geographically may potentially help to reduce health inequalities.

## Highlights

• Our study demonstrates a gradient for arts engagement across area deprivation.
• Arts engagement varies based on neighbourhood characteristics.
• Spatial setting has less influence on levels of arts engagement.
• Interactions are found between individual and geographical factors and engagement.

## Introduction

Over the past two decades, more than 3000 studies have identified the positive impact of arts participation (actively engaging in arts activities such as music, dance or crafts) and cultural engagement (visiting cultural venues or heritage sites) on mental and physical health outcomes.^1^ However, despite growing public awareness of the benefits of engagement and increased promotion of such activities within health care, including through direct referrals from healthcare professionals to arts activities, arts participation and cultural engagement (hereafter referred to collectively as ‘arts engagement’) remains uneven.^2^

To date, much research into factors affecting arts engagement has focused on individual-level characteristics. For example, studies have highlighted how arts engagement is socially patterned, with people of higher socio-economic status (SES) being more likely to engage in the arts.^3^, ^4^, ^5^ One explanation for this is that people's engagement may be influenced by monetary resources, acquired tastes and cultural exclusion.^6^ Furthermore, previous research has suggested that there are gender and ethnic differences in arts engagement, with women^7^^,^^8^ and individuals who are part of the ethnic majority more likely to engage in these activities.^9^

However, arts engagement may also be shaped by geographical factors.^10^^,^^11^ There is evidence that individual social, economic and behavioural outcomes are associated with neighbourhood conditions.^12^ This stems from the concept of ‘neighbourhood effects’, which posits that factors such as environmental conditions, social processes, transportation and other local characteristics can directly and indirectly influence individuals' behaviours.^11^^,^^12^ Therefore, it has been argued that neighbourhood risk factors in concert with individual characteristics can explain a larger proportion of individual behaviours than merely focussing on individual characteristics alone.^12^ In considering how this occurs, researchers have proposed several theoretical mechanisms, two of which are particularly relevant to this study. First, geographical variations in arts engagement could be due to the characteristics of places (such as number of arts venues, arts programmes, studios) shaping people's behaviours.^11^ Arts funding is not geographically equal across countries. For example, in England, funding from Arts Council England (ACE) is greatest in London and lowest in the South West,^13^ even though the South West has one of the highest arts participation rates across England.^14^ Second, spatial differences in population composition such as the socio-economic characteristics of individuals living in an area may lead to collective behavioural influences on arts engagement.^3^^,^^11^^,^^15^ Population composition has been found to influence a number of health behaviours such as food consumption,^16^ smoking,^17^ alcohol consumption^18^ and physical activity.^19^ But to date, very little research has been conducted to understand whether similar place-based effects exist for arts engagement.^11^^,^^20^

Examining the geographical patterns of arts engagement has particular policy relevance at present. First, the well-established evidence for the health benefits of arts engagement has attracted doctors to adopt ‘social prescribing’ schemes in many Western countries, including the UK,^21^, ^22^, ^23^ North America^24^ and Scandinavia.^25^ Social prescribing is a place-based approach that is designed to improve well-being by putting patients in contact with local community activities. However, the importance of ‘place’ in affecting behavioural engagement with the arts is as yet unknown. Second, there is increasing interest in place-based funding to help improve the cultural capacity and capital of an area (particularly areas of high deprivation). For example, the ‘Creative People and Places’ funded by ACE offers financial support to arts-related projects in areas where involvement in arts and cultural activities is below the national average.^26^ But it is unclear if and how the characteristics of the places chosen could affect individual behaviours. A fuller understanding of the relationship between geographical factors and arts engagement is therefore crucial for yielding useful information for public health action.

Therefore, in this article, we used a large nationally representative sample of adults living in the UK to examine whether both arts participation and cultural engagement vary geographically. We examined two main types of geographical factors: (i) the broad spatial setting as an indicator of geographical contexts, measured by regional locations within England and the urbanisation of areas, and (ii) neighbourhood characteristics as an indicator of local population composition, measured by the level of area deprivation and a geodemographic area classification. Furthermore, given that residential sorting means there is often a close association between place characteristics on the one hand and individual demographic and SES characteristics on the other (e.g. highly educated people are likely to live in wealthier areas, while poorer households can only afford less affluent places), we further explored the extent to which the association between geographical factors and arts engagement is moderated by individual demographic and socio-economic characteristics.

## Methods

We used data from Understanding Society; a large nationally representative UK household longitudinal survey.^27^ Understanding Society follows over 50,000 individuals from over 30,000 households annually. The survey contains a rich set of variables including education, employment, social engagement and health. One attraction of Understanding Society is that participating households’ addresses have been matched to the Office for National Statistics Postcodes Directory and then geocoded into a range of zoning systems (e.g. local government, health and census geographies). In this study, we used data from the Wave two interview (2010/12) which has an overall sample of 38,069. In our analysis, we included participants who provided full data across all measures (*N* = 26,215).

### Measures

In a previous study, we applied latent class analysis (LCA) to the same sample used in this study and identified two patterns of participation in arts activities and engagement with culture and heritage.^2^ Arts participation was made up of five items (including playing a musical instrument and painting, drawing, printmaking or sculpture), whereas cultural engagement was made up of 14 items (including visited an art or craft exhibition, visited a musical or dancing performance, visited museums and heritage sites). A full list of the activities of arts participation and cultural engagement can be found in Appendix A. Respondents were asked whether or not they had engaged in each of the arts/cultural activities in the past 12 months.

For arts participation, the LCA identified four profiles of engagement: ‘engaged omnivores’, who took part in lots of activities (1.18% of our sample); people who mainly participated in either ‘visual and literary arts’ (4.54%) or ‘performing arts’ (4.63%) and people who were largely ‘disengaged’ (89.7%).^2^ To ensure a more balanced sample between the groups, the sample was split into ‘engaged’ (which included individuals in the engaged omnivores, visual and literary arts and performing arts classes) vs ‘disengaged’ (those in the original disengaged class). For cultural engagement, LCA identified three profiles of engagement: ‘rarely engaged’, ‘infrequently engaged’ and ‘frequently engaged’. For full details of the construction of the LCAs, refer our previous article.^2^

For geographical factors, we used each household's Lower Layer Super Output Area (LSOA) identification code. LSOAs are small spatial units used for the release of English and Welsh census data that are designed to be relatively socially homogenous and with boundaries that follow topographical features such as roads or railways. Four geographical variables were considered: rural-urban classification which indicates LSOA level of urbanisation (rural town and fringe vs rural village vs urban city and town vs urban conurbation); regions which include North (North East, North West and Yorkshire and the Humber), Midlands (East and West Midlands) and South (East, London, South East and South West) within England; Index of Multiple Deprivation (IMD 2015), which measures the relative deprivation for small areas according to various domains (e.g. income deprivation, living environment deprivation, crime; measured as most deprived 10% vs medium vs least deprived 10%) and the geodemographic Output Area Classification (OAC), which places LSOAs into 8 super groups on the basis of cluster analysis conducted using a swathe of standardised 2011 census variables (including demographic structure, household composition, housing, employment, SES and population density).^28^ The OAC categorises LSOAs into cosmopolitan student neighbourhoods vs countryside living vs ethnically diverse professionals vs hard-pressed communities vs inner city cosmopolitan vs multicultural living vs suburban living vs industrious communities.

To isolate the independent associations between geographical factors and arts engagement, we also considered a range of individual- and household-level demographic and socio-economic predictors. Demographic factors included respondents’ age, gender, ethnicity, whether the respondent was living alone, partnership status and whether respondents were responsible for children aged 16 years and younger. Socio-economic characteristics included educational attainment, current employment status and occupational SES, parental SES when respondents were aged 14 years, logged monthly household income and housing tenure. A full list of the predictors can be found in our previous study.^2^

### Statistical analysis

To understand the associations between geographical factors and arts engagement, we used binary logistic regression models for arts participation and ordinal logistic regression models for cultural engagement. All analyses were weighted to account for non-response and uneven selection probabilities using the wave 2 cross-sectional weights supplied with Understanding Society.^29^ Odds ratios (ORs) are presented to show the likelihood that an individual would belong to a certain outcome class relative to a given baseline scenario. An OR that is greater than one indicates a higher likelihood of being in the ‘engaged’ groups, while an OR that is lower than one suggests a lower likelihood of being in these groups. Given that arts participation and cultural engagement are likely to be associated at the household level (e.g. a person is likely to participate in arts activities if their family also engages in such activities), the 95% confidence interval (CI) in regression models was calculated by clustering standard errors within households.

In our main analyses, we ran four sets of models each for arts participation and for cultural engagement. The first models examined the association between geographical factors and arts and cultural activities by testing each of the factors individually. In our second model, we examined the association by including all geographical factors simultaneously in one model. To avoid standard errors being inflated (i.e. the 95% CI of the estimates getting too wide) by the inclusion of multiple geographical variables measuring similar things, we only included the measures with the strongest relationship to arts engagement in this simultaneous model and also in subsequent analyses. To understand whether the association between geographical factors and participation could be explained by individual demographic and socio-economic characteristics, the third model additionally included individual demographic factors, while the fourth model added individual SES controls.

In addition to our main analyses, we estimated several alternative specifications as robustness checks; results are presented in the online supplementary material. As an initial check, we reran the analyses by (1) omitting OAC and (2) omitting IMD from the models to assess whether the estimates were affected by including these two potentially collinear variables simultaneously. In addition, to assess whether geographical factors were associated with a certain type of arts participation engagers, we carried out analyses by using the four-fold category (i.e. ‘engaged omnivore’, visual and literary arts', ‘performing arts’ and ‘disengaged’) identified in our prior LCA^2^ and by using multinomial logistic regression (relative risk ratios are presented). Finally, we performed several interaction analyses to test whether the effects of individual characteristics vary across places.

## Results

### Demographics

The average age of our sample was 48 years (SD = 18.4), 55% were women and 91% were white. The distribution of arts participation and cultural engagement groups by demographic backgrounds and socio-economic characteristics was presented in our previous study.^2^ Descriptive statistics showing the distribution of arts participation and cultural engagement by geographical factors are shown in Supplementary Tables 1 and 2

### Geographical factors and arts and cultural engagement

#### Arts participation

Regarding spatial setting and using the ‘disengaged’ as the reference group, respondents who lived in Northern England or the Midlands were less likely to engage in arts activities than those who lived in the South (Table 1). However, there was no association between urbanisation and arts participation.

**Table 1:** Logistic regressions estimating the relationship between geographical factors and arts participation: each geographical factor is included in individual models (weighted; *N* = 26,215).

Geographical factors	Engaged vs disengaged		
	OR	95% CI	*P*-value
***Spatial setting***			
*Model 1 Rural-urban classification only*			
Rural town and fringe	0.98	0.84–1.14	0.753
Rural village	1.15	0.98–1.35	0.080
Urban conurbation	1.00	0.90–1.12	0.994
(ref: Urban city and town)			
**Pseudo R2**	**0.0002**		
*Model 2 Regions only*			
North (North East, North West and Yorkshire and the Humber)	0.77	0.69–0.86	0.000
Midlands (East Midlands and West Midlands)	0.82	0.73–0.93	0.002
(ref: South (London, South East, South West and East))			
**Pseudo R2**	**0.0020**		
***Neighbourhood characteristics***			
*Model 3 Index of Multiple Deprivation only*			
Least deprived 10%	1.21	1.05–1.40	0.007
Most deprived 10%	0.83	0.69–1.01	0.057
(ref: Medium)			
**Pseudo R2**	**0.0009**		
*Model 4 Output Area Classification only*			
Cosmopolitan student neighbourhoods	2.23	1.75–2.85	0.000
Countryside living	1.32	1.13–1.54	0.000
Ethnically diverse professionals	1.27	1.09–1.49	0.002
Hard-pressed communities	0.89	0.75–1.05	0.173
Inner city cosmopolitan	1.91	1.48–2.46	0.000
Multicultural living	1.00	0.83–1.21	0.964
Suburban living	1.10	0.95–1.27	0.222
(ref: Industrious communities)			
**Pseudo R2**	**0.0075**		

The bold values indicate Pseudo R2, which is a measure of how well variables of the model explain the arts engagement.

CI, confidence interval; OR, odds ratio.

Regarding neighbourhood characteristics, respondents living in the 10% least deprived of LSOAs had a 21% higher odds of engaging in the arts, compared with those who were living in areas of a medium level of deprivation, and there was an indication that individuals living in the 10% most deprived areas had a lower odds of engaging in the arts (*P* = 0.057). Compared with people in industrious communities, those who lived in cosmopolitan student neighbourhoods had a 2.2 times higher odds of engaging in the arts. Those who lived in areas designated as countryside living, ethnically diverse professionals and inner city cosmopolitan had 32%, 27% and 91% higher odds of engaging in the arts, respectively. But there was no difference in participation amongst those living in hard-pressed communities or suburban or multicultural LSOAs.

When considering all geographical factors simultaneously, the associations between regions, IMD and OAC and arts participation remained (Table 2) (NB rural-urban classification was removed due to its limited effects and to avoid multicollinearity with OAC). Adjusting for demographic characteristics did not lead to attenuation of the findings. When adjusting for individual socio-economic factors, however, many results were attenuated. The only spatial differences that remained were a 14% lower odds of participating in the arts amongst those in the North than those in the South and a 19% higher odds of engaging amongst those living in the countryside than those living in industrious communities.

**Table 2:** Logistic regressions estimating the relationship between geographical factors and arts participation (weighted; *N* = 26,215).

Geographical factors	Unadjusted			Adjusted for demographic factors			Adjusted for demographic and socio-economic factors		
	Engaged vs disengaged			Engaged vs disengaged			Engaged vs disengaged		
	OR	95% CI	*P*-value	OR	95% CI	*P*-value	OR	95% CI	*P*-value
***Spatial setting***									
*Regions*									
North (North East, North West and Yorkshire and the Humber)	0.87	0.77–0.98	0.022	0.85	0.75–0.95	0.006	0.86	0.76–0.96	0.011
Midlands (East Midlands and West Midlands)	0.92	0.81–1.04	0.166	0.90	0.79–1.02	0.109	0.95	0.84–1.09	0.472
(ref: South (London, South East, South West and East))									
***Neighbourhood characteristics***									
*Index of Multiple Deprivation*									
Least deprived 10%	1.30	1.11–1.53	0.001	1.34	1.14–1.58	0.000	1.15	0.98–1.36	0.085
Most deprived 10%	0.97	0.78–1.22	0.810	0.97	0.78–1.21	0.780	1.15	0.92–1.44	0.217
(ref: Medium)									
*Output Area Classification*									
Cosmopolitan student neighbourhoods	2.17	1.70–2.78	0.000	1.66	1.29–2.13	0.000	1.20	0.92–1.56	0.174
Countryside living	1.27	1.09–1.48	0.003	1.35	1.15–1.58	0.000	1.19	1.02–1.40	0.032
Ethnically diverse professionals	1.18	1.01–1.38	0.040	1.14	0.98–1.34	0.098	0.99	0.84–1.16	0.901
Hard-pressed communities	0.91	0.75–1.10	0.319	0.83	0.68–1.00	0.052	0.98	0.81–1.19	0.831
Inner city cosmopolitan	1.79	1.38–2.32	0.000	1.51	1.15–2.00	0.003	1.20	0.91–1.57	0.193
Multicultural living	0.98	0.80–1.19	0.828	0.90	0.73–1.13	0.367	0.87	0.70–1.09	0.224
Suburban living	0.98	0.83–1.15	0.788	0.99	0.84–1.17	0.912	0.89	0.75–1.05	0.164
(ref: Industrious communities)									
**Pseudo R2**	**0.0089**			**0.0365**			**0.0770**		

Note: Demographic factors include respondents' age, gender, ethnicity, whether or not living alone, partnership status and whether or not responsible for children under age 16. Socio-economic factors include educational level, SES, parental SES, monthly household income and housing tenure. The bold values indicate Pseudo R2, which is a measure of how well variables of the model explain the arts engagement.

CI, confidence interval; OR, odds ratio; SES, socio-economic status.

#### Cultural engagement

Regarding spatial setting, respondents who lived in rural town and fringe and rural villages had higher odds of being culturally engaged than those who lived in urban areas (Table 3). In contrast, people who lived in urban conurbations were less likely to engage in cultural activities. Compared with people living in the South, those residing in the North and Midlands had a lower propensity to be culturally engaged (North: OR = 0.80, 95% CI = 0.75–0.86; Midlands: OR = 0.78, 95% CI = 0.72–0.84) (Table 4).

**Table 3:** Ordinal logistic regressions estimating the relationship between geographical factors and cultural engagement: each geographical factor is included in individual models (weighted; *N* = 26,215).

Geographical factors	Cultural engagement (rarely engaged, infrequently engaged, frequently engaged)		
	OR	95% CI	*P*-value
***Spatial setting***			
*Model 1 Rural-urban classification only*			
Rural town and fringe	1.17	1.06–1.29	0.002
Rural village	1.34	1.20–1.49	0.000
Urban conurbation	0.93	0.87–1.00	0.049
(ref: Urban city and town)			
Cut 1	0.79	0.75–0.82	
Cut 2	3.94	3.76–4.14	
**Pseudo R2**	**0.0015**		
*Model 2 Regions only*			
North (North East, North West and Yorkshire and the Humber)	0.80	0.75–0.86	0.000
Midlands (East Midlands and West Midlands)	0.78	0.72–0.84	0.000
(ref: South (London, South East, South West and East))			
Cut 1	0.69	0.66–0.73	
Cut 2	3.48	3.31–3.66	
**Pseudo R2**	**0.0019**		
***Neighbourhood characteristics***			
*Model 3 Index of Multiple Deprivation only*			
Least deprived 10%	2.01	1.82–2.21	0.000
Most deprived 10%	0.36	0.32–0.41	0.000
(ref: Medium)			
Cut 1	0.76	0.74–0.79	
Cut 2	3.97	3.80–4.14	
**Pseudo R2**	**0.0172**		
*Model 4 Output Area Classification only*			
Cosmopolitan student neighbourhoods	2.11	1.74–2.56	0.000
Countryside living	1.52	1.38–1.68	0.000
Ethnically diverse professionals	1.41	1.28–1.56	0.000
Hard-pressed communities	0.52	0.47–0.57	0.000
Inner city cosmopolitan	1.65	1.34–2.01	0.000
Multicultural living	0.56	0.49–0.63	0.000
Suburban living	1.51	1.38–1.65	0.000
(ref: Industrious communities)			
Cut 1	0.84	0.79–0.90	
Cut 2	4.46	4.17–4.76	
**Pseudo R2**	**0.0240**		

The bold values indicate Pseudo R2, which is a measure of how well variables of the model explain the arts engagement.

CI, confidence interval; OR, odds ratio.

**Table 4:** Ordinal logistic regressions estimating the relationship between geographical factors and cultural engagement (weighted; *N* = 26,215).

Geographical factors	Cultural engagement (rarely engaged, infrequently engaged, frequently engaged)								
	Unadjusted			Adjusted for demographic factors			Adjusted for demographic and socio-economic factors		
	OR	95% CI	*P*-value	OR	95% CI	*P*-value	OR	95% CI	*P*-value
***Spatial setting***									
*Regions*									
North (North East, North West and Yorkshire and the Humber)	0.98	0.91–1.06	0.611	0.97	0.90–1.04	0.363	1.02	0.95–1.10	0.617
Midlands (East Midlands and West Midlands)	0.87	0.80–0.94	0.001	0.86	0.80–0.94	0.000	0.96	0.88–1.04	0.322
(ref: South (London, South East, South West and East))									
***Neighbourhood characteristics***									
*Index of Multiple Deprivation*									
Least deprived 10%	1.78	1.60–1.98	0.000	1.80	1.61–2.01	0.000	1.45	1.29–1.62	0.000
Most deprived 10%	0.56	0.49–0.64	0.000	0.58	0.50–0.66	0.000	0.79	0.69–0.91	0.001
(ref: Medium)									
*Output Area Classification*									
Cosmopolitan student neighbourhoods	2.11	1.74–2.56	0.000	2.21	1.82–2.68	0.000	1.87	1.53–2.28	0.000
Countryside living	1.47	1.33–1.63	0.000	1.45	1.31–1.61	0.000	1.18	1.07–1.31	0.001
Ethnically diverse professionals	1.30	1.18–1.44	0.000	1.33	1.20–1.48	0.000	1.03	0.92–1.14	0.640
Hard-pressed communities	0.64	0.57–0.72	0.000	0.64	0.57–0.72	0.000	0.88	0.78–1.00	0.042
Inner city cosmopolitan	1.68	1.37–2.07	0.000	2.08	1.68–2.57	0.000	1.65	1.36–2.00	0.000
Multicultural living	0.62	0.55–0.70	0.000	0.83	0.73–0.95	0.009	0.86	0.75–0.99	0.032
Suburban living	1.21	1.10–1.34	0.000	1.19	1.08–1.32	0.001	0.93	0.84–1.03	0.144
(ref: Industrious communities)									
Cut 1	0.81	0.75–0.87		0.37	0.32–0.43		1.89	1.15–3.11	
Cut 2	4.35	4.02–4.69		2.09	1.81–2.41		14.01	8.51–23.06	
**Pseudo R2**	**0.0302**			**0.0465**			**0.1328**		

Note: Demographic factors include respondents' age, gender, ethnicity, whether or not living alone, partnership status and whether or not responsible for children aged younger than 16 years. Socio-economic factors include educational level, SES, parental SES, monthly household income and housing tenure. The bold values indicate Pseudo R2, which is a measure of how well variables of the model explain the arts engagement.

CI, confidence interval; OR, odds ratio; SES, socio-economic status.

Regarding neighbourhood characteristics, compared with people living in areas of medium deprivation, those who lived in the 10% least deprived areas had 2 times the odds of more likely being culturally engaged. People who lived in the 10% most deprived areas had a lower propensity to be culturally engaged (OR = 0.36, 95% CI = 0.32–0.41). Compared with individuals residing in industrious communities, cultural engagement is more frequent amongst people living in cosmopolitan student neighbourhoods (2.1 times higher odds), the countryside (1.5 times higher odds), areas of ethnically diverse professionals (1.4 times higher odds), inner city cosmopolitan areas (1.7 times higher odds) and suburban LSOAs (1.5 times higher odds). Conversely, people who lived in hard-pressed communities and LSOAs designated ‘multicultural living’ had a lower propensity to be culturally engaged (hard-pressed communities: OR = 0.52, 95% CI = 0.47–0.57; multicultural living: OR = 0.56, 95% CI = 0.49–0.63).

When considering all geographical factors simultaneously, the relationship for regions was attenuated, with just the Midlands showing lower participation than the South, although other findings remained. There was no attenuation when adjusting for individual demographic factors. When additionally adjusting for individual socio-economic factors, the relationship for regions was completely attenuated. However, the findings for deprivation remained. Furthermore, compared with people who lived in industrious communities, areas of cosmopolitan student neighbourhoods, countryside living and inner city cosmopolitan LSOAs still had higher odds of being culturally engaged, while people living in hard-pressed communities and areas of multicultural living still had a lower odds of being culturally engaged.

### Sensitivity analyses

Results were not significantly different when IMD and OAC were omitted from the models (Supplementary Tables 3a and 3b). When using the four-factor model for different patterns of arts participation rather than a binary measure of engagement vs disengagement, lower participation amongst individuals living in the North was found for general engagement, visual and literary arts engagement and performing arts engagement, but there was lower engagement in the Midlands only for visual and literary arts engagement (Supplementary Tables 4a and 4b). Area deprivation appeared most important in relation to performing arts, as did living in the countryside. Living in a hard-pressed community was strongly related to a lower odds of being an engaged omnivore. There were no other major differences depending on the type of arts participation.

Finally, there were no interactions between individual and geographical factors for arts participation (results not shown). However, the positive associations between higher SES and education and cultural engagement are more pronounced in highly deprived areas and less pronounced in more affluent places (Supplementary Table 5a -5c).

## Discussion

While there is evidence that there is a social gradient across arts participation and cultural engagement, this study explores how patterns of arts engagement are also geographically patterned across the UK. Importantly, it extends existing studies which largely presented evidence on regional differences in arts engagement by assessing how both broad spatial setting and local neighbourhood characteristics may help explain these geographic divides.

Our results demonstrate that there are geographical differences in participation even after controlling for individual demographic and socio-economic backgrounds. In particular, individuals living in the North of England had lower odds of engaging in the arts, while those living in the countryside had higher odds, especially for engaging in performing arts activities. For cultural engagement, the geographical region was less important, but area deprivation predicted patterns of engagement; engagement was also higher amongst those living in cosmopolitan student neighbourhoods and in relatively affluent countryside areas but lower amongst those living in hard-pressed communities. In addition for cultural engagement, individuals who had a disjunction between their own SES and education levels and the level of deprivation where they lived had higher engagement.

Overall, there was some evidence that spatial setting predicted arts engagement. Rural-urban classification made no difference to arts participation, but did predict cultural engagement, with higher engagement in rural settings. Although museums are distributed across both urban and rural settings, many heritage sites are located in rural settings,^30^ and so it is possible that local availability of cultural assets drives these differences. Geographical region did predict both arts participation and cultural engagement, with lower engagement in arts participation in the North and in cultural engagement in the Midlands than in Southern England. The finding for arts participation echoes government reports on participation.^14^ However, for cultural engagement, this relationship was explained away by individual socio-economic factors, suggesting that region itself was less important than the wealth and education of individuals living within it.

There was much more evidence for differences in arts engagement based on neighbourhood characteristics. For arts participation, living in a less deprived area was associated with greater engagement, but this was explained away by individual SES, and there was only limited evidence that living in certain types of neighbourhoods (i.e. countryside living) predicted engagement. However, for cultural engagement, neighbourhood deprivation remained a significant predictor, as did living in particular types of neighbourhoods, especially those that were cosmopolitan or in the countryside. This suggests that the collective behaviours of individuals living in the community, as well as sorting into neighbourhoods on the basis of cultural preferences, could be important predictors of cultural engagement.

Borrowing the model of ‘food environment’ and health,^16^ which explores how neighbourhood characteristics influence food consumption, three factors are key: (i) the availability of arts/cultural facilities, events and programmes in one's locale; (ii) the accessibility to the location of where arts/cultural activities are provided and the ease of getting to the location; and (iii) the affordability in terms of monetary resources to people living in the catchment area. These three elements are usually found in areas that are characterised as cosmopolitan, culturally developed, have lower levels of area deprivation and possibly have strong social ties within the neighbourhood (especially in rural areas);^11^^,^^16^ precisely the three areas where we found higher levels of cultural engagement. Alternatively, people who live in hard-pressed communities, where the rate of unemployment is relatively higher and education level is lower, or areas of multicultural living where a high proportion of residents are non-white, may have a lower participation rate due to the inadequacy of these three elements. This finding echoes previous studies on the negative association between individual SES and ethnic minorities and arts and cultural participation^3^, ^4^, ^5^^,^^9^^,^^31^ but extends these findings by showing how the impact of people with these characteristics living in a particular area could have a collective influence on behaviours, irrespective of individual characteristics.

It is also notable that we found some evidence of ‘disjunction’: individuals living in areas where their own material circumstances were at odds with the level of deprivation around them had higher patterns of engagement. Notably this went both ways (i.e. higher education/wealth in a more deprived area and lower education/wealth in a more affluent area). This suggests that whilst neighbourhood characteristics are important, they are not deterministic. It is possible that where individuals perceive a disjunction (i.e. people of higher educational attainment living in a more deprived area), they may still want to cultivate cultural tastes and preferences by engaging in the activities regardless of where they live.^32^ Thus, they may make a specific effort to engage in cultural activities, for example, to display their gentrifying credentials. What remains unclear, however, is whether this engagement involves them staying within their home neighbourhoods or seeking cultural activities deliberately outside of their neighbourhood. It is also plausible that for people of lower educational attainment, the availability of recreational infrastructure (e.g. arts exhibitions/theatres) and the local conditions (e.g. the perceived neighbourhood safety) which are usually found in affluent areas could encourage arts engagement.

This study has a number of strengths including using a nationally representative sample and a rich set of variables to map comprehensively individuals’ engagement in arts and cultural activities. Furthermore, we used four different geographical factors assessing both spatial setting and neighbourhood characteristics. However, there are also several weaknesses. For instance, while we identified the patterns of arts and cultural engagement across the country, we were unable to distinguish people who lacked opportunities to engage from those who were disinterested in engaging. Furthermore, the relationship between geographical factors and engagement may be affected by self-selection biases: people may choose to live in areas where there are more opportunities for arts and cultural engagement (e.g. cosmopolitan cities) because this is a particular interest for them. As such, our present study is not able to identify whether geography causally influences behaviours or whether personal interests play a role in residential selection. Future studies could examine whether the link between arts and health varies across locations, that is, whether geographical factors not only predict engagement but also act as a moderator for the health benefits of engagement.

Overall, this study found that arts participation and cultural engagement are associated with geographical factors independent of individuals' observed demographic and socio-economic backgrounds. In particular, it goes beyond describing regional variations in patterns of engagement to show that neighbourhood characteristics may be a stronger predictor for engagement behaviour than spatial setting and may have a greater relationship with cultural engagement than with arts participation. Understanding the role of geography in arts engagement is relevant to public health initiatives and interventions (e.g. the roll out of ‘social prescribing’ scheme delivered in various western countries) as it has the potential to increase engagement through redistributing resources (e.g. funding and cultural facilities) to various areas and providing opportunities to engage. Given the benefits of the arts, place-based interventions could help improve health and well-being on a population level by reaching individuals who are at risk of poor health/well-being and who have traditionally been excluded from artistic experiences due to geographical barriers. This could potentially help reduce health inequalities through equalising access to arts and cultural programmes.^33^ However, this remains to be tested further through intervention place-based studies. Nevertheless, our study suggests the importance of considering geographical as well as individual-level predictors when devising policies to improve access to and engagement with the arts.

## Author statements

### Ethical approval

The University of Essex Ethics Committee has approved all data collection on Understanding Society main study and innovation panel waves, including asking consent for all data linkages except to health records. Respondents aged 16 years or older provided written consent to participate.

### Funding

D.F. is supported by the Wellcome Trust [205407/Z/16/Z]. H.W.M. is funded through the 10.13039/501100000267AHRC project HEARTS [AH/P005888/1]. This project is also supported by UCL Grand Challenges Award ‘Environment and Wellbeing’ [551987/100/156425 33002], the 10.13039/501100000275Leverhulme Trust [PLP-2018-007] and 10.13039/501100000269ESRC WELLCOMM project [ES/T006994/1].

### Competing interests

The authors have no conflicts of interest to disclose.

### Author contributions

H.W.M. conducted the data management and data analyses and provided input on the manuscript. R.C. and D.F. assisted with analytical issues and provided input on the analytical scheme and the manuscript. All authors are responsible for reported research and have participated in the concept and design, analysis and interpretation of data and drafting and revising of the manuscript.

### Availability of data and materials

Understanding Society - The UK Household Longitudinal Study (UKHLS) data are available from the UK Data Service https://discover.ukdataservice.ac.uk/catalogue/?sn = 6614. Data documentation is available from the Understanding Society website https://www.understandingsociety.ac.uk/documentation.
