# Supplementary material for: Does arts and cultural engagement vary geographically? Evidence from the UK household longitudinal study
Source: Public Health. 2020 Aug;185:119–26. doi: 10.1016/j.puhe.2020.04.029 (PMC7456771; doi:10.1016/j.puhe.2020.04.029)
Supplement: Multimedia component 1 [file mmc1.docx]

**Does arts and cultural engagement vary geographically? Evidence from the UK Household Longitudinal Study**

**Supplementaries**

***Appendix A A list of activities of arts participation and cultural engagement***

*Arts participation*

- Sang to an audience or rehearsed for a performance (not karaoke)
- Played a musical instrument
- Rehearsed or performed in a play/drama, opera/operetta or musical theatre
- Painting, drawing, printmaking or sculpture
- Written any stories, plays or poetry

*Cultural engagement*

- Been a member of a book club, where people meet up to discuss and share books/ been to an event connected with books or writing
- Been to an exhibition or collection of art, photography or sculpture or a craft exhibition (not craft market)
- Been to a play/drama, pantomime or a musical
- Been to an opera/operetta or a classical music performance
- Been to a rock, pop or jazz performance
- Been to ballet or contemporary dance
- Visited museums
- Visited a city or town with historic character
- Visited a historic building open to the public (non-religious)
- Visited a place connected with industrial history (e.g. an old factory, dockyard or mine) or historic transport system (e.g. an old ship or railway)
- Visited a historic place of worship attended as a visitor (not to worship)
- Visited a monument such as a castle, fort or ruin
- Visited a site of archaeological interest (e.g. Roman villa, ancient burial site)
- Visited a site connected with sports heritage (e.g. Wimbledon) (not visited for the purposes of watching sport)

| **Supplementary Table 1 Distribution of arts participation groups by geographical factors % (weighted; N=38,069)** | | | |
| --- | --- | --- | --- |
|  | **Engaged** | **Disengaged** | **Total** |
| **Relative size (%)** | **10.3** | **89.7** | **100** |
| ***Spatial setting*** |  |  |  |
| *Rural-Urban Classification* |  |  |  |
| Rural town and fringe | 9.74 | 9.77 | 9.77 |
| Rural village | 8.93 | 7.92 | 8.03 |
| Urban city and town | 44.4 | 44.5 | 44.5 |
| Urban conurbation | 36.9 | 37.8 | 37.7 |
| *Regions* |  |  |  |
| North (North East, North West and Yorkshire and the Humber) | 24.3 | 28.8 | 28.3 |
| Midlands (East Midlands and West Midlands) | 17.6 | 19.3 | 19.1 |
| South (London, South East, South West and East) | 58.1 | 52.0 | 52.6 |
| ***Neighbourhood characteristics*** |  |  |  |
| *Index of Multiple Deprivation* |  |  |  |
| Least deprived 10% | 11.8 | 9.76 | 9.98 |
| Medium | 81.0 | 81.2 | 81.2 |
| Most deprived 10% | 7.20 | 9.05 | 8.85 |
| *Output Area Classification* |  |  |  |
| Cosmopolitan student neighbourhoods | 5.13 | 2.65 | 2.92 |
| Countryside living | 14.3 | 12.6 | 12.8 |
| Ethnically diverse professionals | 16.8 | 15.6 | 15.8 |
| Hard-pressed communities | 10.1 | 13.3 | 13.0 |
| Industrious communities | 18.3 | 21.1 | 20.8 |
| Inner city cosmopolitan | 8.79 | 5.58 | 5.93 |
| Multicultural living | 9.41 | 11.4 | 11.2 |
| Suburban living | 17.1 | 17.8 | 17.7 |

| **Supplementary Table 2 Distribution of cultural engagement groups by geographical factors % (weighted; N=38,069)** | | | | |
| --- | --- | --- | --- | --- |
|  | **Rarely engaged** | **Infrequently engaged** | **Frequently engaged** | **Total** |
| **Relative size** | **47.7** | **33.9** | **18.4** | **100** |
| ***Spatial setting*** |  |  |  |  |
| *Rural-Urban Classification* |  |  |  |  |
| Rural town and fringe | 8.72 | 10.6 | 10.7 | 9.77 |
| Rural village | 6.80 | 8.44 | 10.1 | 8.03 |
| Urban city and town | 44.3 | 45.7 | 42.9 | 44.5 |
| Urban conurbation | 40.2 | 35.3 | 36.4 | 37.7 |
| *Regions* |  |  |  |  |
| North (North East, North West and Yorkshire and the Humber) | 29.8 | 28.0 | 25.3 | 28.3 |
| Midlands (East Midlands and West Midlands) | 20.2 | 19.4 | 15.9 | 19.1 |
| South (London, South East, South West and East) | 50.0 | 52.5 | 58.8 | 52.6 |
| ***Neighbourhood characteristics*** |  |  |  |  |
| *Index of Multiple Deprivation* |  |  |  |  |
| Least deprived 10% | 6.09 | 11.4 | 16.2 | 9.98 |
| Medium | 80.4 | 82.2 | 81.0 | 81.2 |
| Most deprived 10% | 13.5 | 6.37 | 2.76 | 8.85 |
| *Output Area Classification* |  |  |  |  |
| Cosmopolitan student neighbourhoods | 1.95 | 3.13 | 4.70 | 2.92 |
| Countryside living | 10.4 | 13.9 | 16.4 | 12.8 |
| Ethnically diverse professionals | 13.5 | 16.8 | 19.0 | 15.8 |
| Hard-pressed communities | 18.0 | 10.9 | 5.38 | 13.0 |
| Industrious communities | 21.3 | 21.6 | 17.9 | 20.8 |
| Inner city cosmopolitan | 5.33 | 4.92 | 9.05 | 5.93 |
| Multicultural living | 15.5 | 8.46 | 6.21 | 11.2 |
| Suburban living | 14.0 | 20.4 | 21.4 | 17.7 |

| **Supplementary Table 3a Estimating the association between Index of Multiple Deprivation and arts and cultural activities while not adjusting for Output Area Classification (weighted; N=26,215)** | | | | | | |
| --- | --- | --- | --- | --- | --- | --- |
|  | **Arts participation (engaged vs disengaged)** | | | **Cultural engagement (rarely engaged, infrequently engaged, frequently engaged)** | | |
|  | **OR** | **95%CI** | **P-value** | **OR** | **95%CI** | **P-value** |
| *Index of Multiple Deprivation* | | |  |  |  |  |
| Least deprived 10% | 1.06 | 0.91-1.23 | 0.447 | 1.33 | 1.20 - 1.48 | 0.000 |
| Most deprived 10% | 1.11 | 0.91-1.35 | 0.309 | 0.72 | 0.64 - 0.82 | 0.000 |
| (ref: Medium) |  |  |  |  |  |  |
| Cut 1 |  |  |  | 1.96 | 1.17 - 3.26 |  |
| Cut 2 |  |  |  | 14.32 | 8.58 - 23.89 |  |
| **Pseudo R2** | **0.0755** |  |  | **0.1292** |  |  |
| Note: Model adjusted for respondents’ age, gender, ethnicity, whether or not living alone, partnership status, whether or not responsible for children under age 16, educational level, SES, parental SES, monthly household income, housing tenure and regions. | | | | | | |

| **Supplementary Table 3b Estimating the association between Output Area Classification and arts and cultural activities while not adjusting for Index of Multiple Deprivation (weighted; N=26,215)** | | | | | | | |
| --- | --- | --- | --- | --- | --- | --- | --- |
|  | **Arts participation (engaged vs disengaged)** | | | | **Cultural engagement (rarely engaged, infrequently engaged, frequently engaged)** | | |
|  | **OR** | | **95%CI** | **P-value** | **OR** | **95%CI** | **P-value** |
| *Output Area Classification* | | | |  |  |  |  |
| Cosmopolitan student neighbourhoods | 1.21 | | 0.93-1.57 | 0.150 | 1.85 | 1.51 - 2.26 | 0.000 |
| Countryside living | 1.20 | | 1.02-1.41 | 0.026 | 1.19 | 1.08 - 1.32 | 0.001 |
| Ethnically diverse professionals | 1.01 | | 0.86-1.18 | 0.939 | 1.05 | 0.94 - 1.16 | 0.379 |
| Hard-pressed communities | 1.03 | | 0.86-1.23 | 0.756 | 0.82 | 0.73 - 0.91 | 0.000 |
| Inner city cosmopolitan | 1.20 | | 0.92-1.58 | 0.181 | 1.57 | 1.29 - 1.90 | 0.000 |
| Multicultural living | 0.89 | | 0.72-1.10 | 0.290 | 0.81 | 0.71 - 0.93 | 0.002 |
| Suburban living | 0.94 | | 0.81-1.10 | 0.449 | 1.06 | 0.96 - 1.16 | 0.243 |
| (ref: Industrious communities) | | | |  |  |  |  |
| Cut 1 | | | |  | 2.01 | 1.22 - 3.32 |  |
| Cut 2 | | | |  | 14.85 | 8.99 - 24.52 |  |
| **Pseudo R2** | | **0.0766** | |  | **0.1313** |  |  |
| Note: Model adjusted for respondents’ age, gender, ethnicity, whether or not living alone, partnership status, whether or not responsible for children under age 16, educational level, SES, parental SES, monthly household income, housing tenure and regions. | | | | | | | |

| **Supplementary Table 4a Multinomial logistic regressions estimating the relationship between geographical factors and four-fold arts participation: each geographical factor is included in individual models (weighted; N=26,215)** | | | | | | | | | | | | | | | | | | | |  |
| --- | --- | --- | --- | --- | --- | --- | --- | --- | --- | --- | --- | --- | --- | --- | --- | --- | --- | --- | --- | --- |
|  | **Engaged omnivore vs disengaged** | | | | | | | | **Visual & literary arts vs disengaged** | | | | | **Performing arts vs disengaged** | | | | | |  |
|  | **RRR** | | | | **95%CI** | | **P-value** | | **RRR** | **95%CI** | | **P-value** | | **RRR** | | **95%CI** | | **P-value** | |  |
| ***Spatial setting*** | | | | | | | | |  |  | |  | |  | |  | |  | |  |
| *Model 1 Rural-Urban Classification only* | | | | | | | | |  |  | |  | |  | |  | |  | |  |
| Rural town and fringe | 1.26 | | | | 0.83-1.91 | | 0.287 | | 0.82 | 0.65-1.03 | | 0.094 | | 1.06 | | 0.86-1.31 | | 0.576 | |  |
| Rural village | 0.66 | | | | 0.39-1.12 | | 0.124 | | 1.04 | 0.82-1.32 | | 0.750 | | 1.40 | | 1.13-1.74 | | 0.002 | |  |
| Urban conurbation | 0.95 | | | | 0.70-1.30 | | 0.770 | | 0.97 | 0.83-1.13 | | 0.668 | | 1.05 | | 0.90-1.22 | | 0.545 | |  |
| (ref: Urban city and town) | | | | | | |  | |  |  | |  | |  | |  | |  | |  |
| **Pseudo R2** | **0.0009** | | | | | |  | |  |  | |  | |  | |  | |  | |  |
| *Model 2 Regions only* | | | | | | |  | |  |  | |  | |  | |  | |  | |  |
| North (North East, North West and Yorkshire and the Humber) | 0.56 | | | | 0.40-0.78 | | 0.001 | | 0.75 | 0.64-0.88 | | 0.000 | | 0.84 | | 0.72-0.98 | | 0.025 | |  |
| Midlands (East Midlands and West Midlands) | 0.84 | | | | 0.59-1.19 | | 0.335 | | 0.70 | 0.59-0.84 | | 0.000 | | 0.94 | | 0.79-1.11 | | 0.443 | |  |
| (ref: South (London, South East, South West and East)) | | | | | | | | | |  | |  | |  | |  | |  | |  |
| **Pseudo R2** | **0.0021** | | | | | | | | |  | |  | |  | |  | |  | |  |
| ***Neighbourhood characteristics*** | | | | | | |  | |  |  | |  | |  | |  | |  | |  |
| *Model 3 Index of Multiple Deprivation only* | | | | | | | | |  |  | |  | |  | |  | |  | |  |
| Least deprived 10% | 1.43 | | | | 0.99-2.08 | | 0.056 | | 0.89 | 0.71-1.10 | | 0.281 | | 1.49 | | 1.24-1.78 | | 0.000 | |  |
| Most deprived 10% | 0.62 | | | | 0.30-1.30 | | 0.207 | | 0.93 | 0.73-1.18 | | 0.531 | | 0.79 | | 0.62-1.01 | | 0.059 | |  |
| (ref: Medium) |  | | | |  | |  | |  |  | |  | |  | |  | |  | |  |
| **Pseudo R2** | **0.0016** | | | |  | |  | |  |  | |  | |  | |  | |  | |  |
| *Model 4 Output Area Classification only* | | | | | | |  | |  |  | |  | |  | |  | |  | |  |
| Cosmopolitan student neighbourhoods | 2.44 | | | | 1.33-4.50 | | 0.004 | | 2.87 | 2.06-3.99 | | 0.000 | | 1.53 | | 1.07-2.19 | | 0.020 | |  |
| Countryside living | 0.81 | | | | 0.51-1.27 | | 0.360 | | 1.19 | 0.95-1.49 | | 0.137 | | 1.62 | | 1.31-2.00 | | 0.000 | |  |
| Ethnically diverse professionals | 0.91 | | | | 0.59-1.39 | | 0.658 | | 1.30 | 1.05-1.61 | | 0.015 | | 1.36 | | 1.09-1.70 | | 0.006 | |  |
| Hard-pressed communities | 0.36 | | | | 0.19-0.71 | | 0.003 | | 1.04 | 0.82-1.32 | | 0.730 | | 0.90 | | 0.70-1.15 | | 0.390 | |  |
| Inner city cosmopolitan | 2.22 | | | | 1.23-4.02 | | 0.008 | | 2.11 | 1.47-3.01 | | 0.000 | | 1.62 | | 1.17-2.24 | | 0.003 | |  |
| Multicultural living | 0.95 | | | | 0.58-1.56 | | 0.834 | | 0.96 | 0.74-1.25 | | 0.767 | | 1.06 | | 0.80-1.41 | | 0.660 | |  |
| Suburban living | 0.95 | | | | 0.64-1.42 | | 0.803 | | 0.89 | 0.72-1.11 | | 0.315 | | 1.35 | | 1.10-1.65 | | 0.004 | |  |
| (ref: Industrious communities) | | | | | | |  | |  |  | |  | |  | |  | |  | |  |
| **Pseudo R2** | | | **0.0086** | | | |  | |  |  | |  | |  | |  | |  | |  |
| **Supplementary Table 4b Multinomial logistic regressions estimating the relationship between geographical factors and four-fold arts participation: all geographical factors, demographics factors and socio-economic factors included in the model (weighted; N=26,215)** | | | | | | | | | | | | | | | | | | | | |
|  | | **Engaged omnivore vs disengaged** | | | | | | **Visual & literary arts vs disengaged** | | | | | | | **Performing arts vs disengaged** | | | | | |
|  | | **RRR** | | **95%CI** | | **P-value** | | **RRR** | | | **95%CI** | | **P-value** | | **RRR** | | **95%CI** | | **P-value** | |
| ***Spatial Setting*** | | | | | | | |  | | |  | |  | |  | |  | |  | |
| *Regions* | | | | | | | |  | | |  | |  | |  | |  | |  | |
| North (North East, North West and Yorkshire and the Humber) | | 0.62 | | 0.43-0.90 | | 0.013 | | 0.80 | | | 0.67-0.94 | | 0.008 | | 0.97 | | 0.83-1.15 | | 0.761 | |
| Midlands (East Midlands and West Midlands) | | 0.99 | | 0.70-1.42 | | 0.976 | | 0.81 | | | 0.67-0.98 | | 0.033 | | 1.08 | | 0.91-1.29 | | 0.383 | |
| (ref: South (London, South East, South West and East)) | | | | | | | | | | | | |  | |  | |  | |  | |
| ***Neighbourhood characteristics*** | | | | | |  | |  | | |  | |  | |  | |  | |  | |
| *Index of Multiple Deprivation* | | | | | | | |  | | |  | |  | |  | |  | |  | |
| Least deprived 10% | | 1.48 | | 0.95-2.30 | | 0.086 | | 0.90 | | | 0.70-1.16 | | 0.407 | | 1.31 | | 1.06-1.62 | | 0.013 | |
| Most deprived 10% | | 1.09 | | 0.46-2.58 | | 0.839 | | 1.17 | | | 0.88-1.55 | | 0.285 | | 1.16 | | 0.88-1.55 | | 0.298 | |
| (ref: Medium) | |  | |  | |  | |  | | |  | |  | |  | |  | |  | |
| *Output Area Classification* | | | | | |  | |  | | |  | |  | |  | |  | |  | |
| Cosmopolitan student neighbourhoods | | 0.89 | | 0.47-1.68 | | 0.727 | | 1.34 | | | 0.95-1.91 | | 0.097 | | 1.03 | | 0.71-1.50 | | 0.877 | |
| Countryside living | | 0.71 | | 0.45-1.14 | | 0.154 | | 1.11 | | | 0.88-1.41 | | 0.360 | | 1.40 | | 1.13-1.74 | | 0.002 | |
| Ethnically diverse professionals | | 0.61 | | 0.39-0.95 | | 0.030 | | 0.99 | | | 0.79-1.24 | | 0.929 | | 1.10 | | 0.87-1.39 | | 0.415 | |
| Hard-pressed communities | | 0.38 | | 0.18-0.81 | | 0.012 | | 1.04 | | | 0.79-1.36 | | 0.789 | | 1.09 | | 0.84-1.43 | | 0.513 | |
| Inner city cosmopolitan | | 1.04 | | 0.57-1.91 | | 0.896 | | 1.15 | | | 0.78-1.71 | | 0.481 | | 1.23 | | 0.87-1.76 | | 0.240 | |
| Multicultural living | | 0.72 | | 0.40-1.31 | | 0.284 | | 0.76 | | | 0.56-1.03 | | 0.076 | | 1.03 | | 0.74-1.45 | | 0.845 | |
| Suburban living | | 0.66 | | 0.41-1.05 | | 0.078 | | 0.83 | | | 0.65-1.05 | | 0.121 | | 1.00 | | 0.80-1.26 | | 0.972 | |
| (ref: Industrious communities) | | | | | |  | |  | | |  | |  | |  | |  | |  | |
| **Pseudo R2** | | | **0.0718** | | |  | |  | | |  | |  | |  | |  | |  | |
| Note: Model adjusted for respondents’ age, gender, ethnicity, whether or not living alone, partnership status, whether or not responsible for children under age 16, educational level, SES, parental SES, monthly household income and housing tenure. | | | | | | | | | | | | | | | | | | | | |

| **Supplementary Table 5a Interaction terms between Index of Multiple Deprivation (least deprived 10% vs medium vs most deprived 10%) and individual SES (higher vs lower) on cultural engagement (N=26,215)** | | | |
| --- | --- | --- | --- |
|  | **Cultural engagement (rarely engaged, infrequently engaged, frequently engaged)** | | |
|  | **OR** | **95%CI** | **P-value** |
| Least deprived 10% | 1.68 | 1.45-1.95 | 0.000 |
| Most deprived 10% | 0.76 | 0.65-0.89 | 0.001 |
| (ref: Medium) |  |  |  |
| Higher SES (intermediate or above) | 1.48 | 1.38-1.59 | 0.000 |
| (ref: Lower SES) |  |  |  |
| *Interaction term between IMD and SES* |  | |  |
| Least deprived 10% * higher SES | 0.76 | 0.64-0.90 | 0.002 |
| Most deprived 10% * higher SES | 1.22 | 0.95-1.55 | 0.115 |
| (ref: Medium and lower SES) |  |  |  |
| Cut 1 | 3.28 | 2.02 - 5.33 |  |
| Cut 2 | 24.20 | 14.88 - 39.38 |  |
| **Pseudo R2** | **0.1319** |  |  |
| Note: Model adjusted for respondents’ age, gender, ethnicity, whether or not living alone, partnership status, whether or not responsible for children under age 16, educational level, parental SES, monthly household income, housing tenure, regions, and output area classification. | | | |

| **Supplementary Table 5b Interaction terms between Index of Multiple Deprivation (least deprived 20% vs medium vs most deprived 20%) and individual SES (higher vs lower) on cultural engagement (N=26,215)** | | | |
| --- | --- | --- | --- |
|  | **Cultural engagement (rarely engaged, infrequently engaged, frequently engaged)** | | |
|  | **OR** | **95%CI** | **P-value** |
| Least deprived 20% | 1.51 | 1.34 - 1.69 | 0.000 |
| Most deprived 20% | 0.77 | 0.67 - 0.88 | 0.000 |
| (ref: Medium) |  |  |  |
| Higher SES (intermediate or above) | 1.49 | 1.37 - 1.61 | 0.000 |
| (ref: Lower SES) |  |  |  |
| *Interaction term between IMD and SES* | |  |  |
| Least deprived 20% * higher SES | 0.80 | 0.70-0.92 | 0.002 |
| Most deprived 20% * higher SES | 1.19 | 1.01-1.42 | 0.040 |
| (ref: Medium and lower SES) |  |  |  |
| Cut 1 | 3.29 | 2.03 - 5.35 |  |
| Cut 2 | 24.26 | 14.91 - 39.49 |  |
| **Pseudo R2** | **0.1321** |  |  |
| Note: Model adjusted for respondents’ age, gender, ethnicity, whether or not living alone, partnership status, whether or not responsible for children under age 16, educational level, parental SES, monthly household income, housing tenure, regions, and output area classification. | | | |

| **Supplementary Table 5c Interaction terms between Index of Multiple Deprivation (least deprived 20% vs medium vs most deprived 20%) and individual educational level (higher vs lower) on cultural engagement (N=26,215)** | | | |
| --- | --- | --- | --- |
|  | **Cultural engagement (rarely engaged, infrequently engaged, frequently engaged)** | | |
|  | **OR** | **95%CI** | **P-value** |
| Least deprived 20% | 1.53 | 1.35 - 1.74 | 0.000 |
| Most deprived 20% | 0.81 | 0.70 - 0.93 | 0.003 |
| (ref: Medium) |  |  |  |
| Higher educational level (A-levels or above) | 2.61 | 2.42 - 2.82 | 0.000 |
| (ref: Lower educational level) |  |  |  |
| *Interaction term between IMD and education* | | |  |
| Least deprived 20% * higher education | 0.84 | 0.73-0.96 | 0.013 |
| Most deprived 20% * higher education | 1.02 | 0.87-1.20 | 0.786 |
| (ref: Medium and lower education) |  |  |  |
| Cut 1 | 6.66 | 3.99 - 11.10 |  |
| Cut 2 | 47.78 | 28.58 - 79.86 |  |
| **Pseudo R2** | **0.1241** |  |  |
| Note: Model adjusted for respondents’ age, gender, ethnicity, whether or not living alone, partnership status, whether or not responsible for children under age 16, SES, parental SES, monthly household income, housing tenure, regions and output area classification. | | | |
